# A Novel Formononetin Derivative Promotes Anti-ischemic Effects on Acute Ischemic Injury in Mice

**DOI:** 10.3389/fmicb.2021.786464

**Published:** 2021-12-14

**Authors:** Lin Zhao, Jing Han, Jiaqi Liu, Kechen Fan, Tianjie Yuan, Ju Han, Liangliang Chen, Sen Zhang, Ming Zhao, Jinao Duan

**Affiliations:** Jiangsu Key Laboratory for High Technology Research of TCM Formulae, Jiangsu Collaborative Innovation Center of Chinese Medicinal Resources Industrialization, Nanjing University of Chinese Medicine, Nanjing, China

**Keywords:** formononetin, succinyl ononin, *Bacillus amyloliquefaciens* FJ18, acute ischemic injury, biotransformation

## Abstract

Natural flavonoids, formononetin and ononin, possess antioxidant, antibacterial, anti-inflammatory and neuroprotective effects. Many complications caused by SARS-CoV-2 make patients difficult to recover. Flavonoids, especially formononetin and ononin, have the potential to treat SARS-CoV-2 and improve myocardial injury. However, their poor water solubility, poor oral absorption, high toxicity, and high-cost purification limit industrial practical application. Succinylation modification provides a solution for the above problems. Formononetin-7-*O*-β-(6″-*O*-succinyl)-D-glucoside (FMP), a new compound, was succinyl glycosylated from formononetin by the organic solvent tolerant bacteria *Bacillus amyloliquefaciens* FJ18 in a 10.0% DMSO (v/v) system. The water solubility of the new compound was improved by over 106 times compared with formononetin, which perfectly promoted the application of formononetin and ononin. The conversion rate of formononetin (0.5 g/L) was almost 94.2% at 24 h, while the yield of formononetin-7-*O*-β-(6″-*O*-succinyl)-D-glucoside could achieve 97.2%. In the isoproterenol (ISO)-induced acute ischemia mice model, the myocardial injury was significantly improved with a high dose (40 mg/kg) of formononetin-7-*O*-β-(6″-*O*-succinyl)-D-glucoside. The lactate dehydrogenase level was decreased, and the catalase and superoxide dismutase levels were increased after formononetin-7-*O*-β-(6″-*O*-succinyl)-D-glucoside treatment. Thus, formononetin-7-*O*-β-(6″-*O*-succinyl)-D-glucoside has high water solubility, low toxicity, and shows significant antimyocardial ischemia effects.

## Introduction

Formononetin (7-hydroxy-4′-methoxyisoflavone) widely distributes in Leguminosae plants, such as *Glycyrrhiza uralensis* Fisch, *Astragalus membranaceus*, *Trifolium pretense*, and *Pueraria lobata* (Wen, 2006; Liu et al., 2007). The antioxidant, hypolipidemic, and cholesterol lowering of formononetin can regulate the blood pressure by adjusting the vascular tension, which contributes to preventing and treating related cardiovascular diseases effectively (Zhao et al., 2009, 2012; Yu et al., 2010). Ononin, referred to as formononetin-7-*O*-β-D-glucoside, is one of the bioactive chemicals found in many functional food or plants (Guowei et al., 2021). Ononin has many pharmacological activities, such as promoting skin growth, scavenging oxygen-free radicals, inhibiting lipid peroxidation, maintaining NO concentration in the blood, and protecting ischemia–reperfusion injury (Wei et al., 2014). Formononetin and ononin have attracted lots of attention due to their outstanding bioactivities and potential medicinal value.

Flavonoids, sharing the basic functional group with estrogen, have been utilized as chemopreventive agents to inhibit endothelial cell angiogenesis, and suppress tumor cell proliferation (Guowei et al., 2021). Myocardial tissue cells are most likely to be infected by SARS-CoV-2, which may directly invade myocardial cells and cause myocardial cell necrosis (Xiao, 2021). Among natural products, flavonoids can be promising SARS-CoV-2 inhibitors (Ibrahim et al., 2021). Especially, formononetin and ononin possess the antimyocardial ischemia effect and have the potential to treat myocardial ischemia caused by SARS-CoV-2; thus, the pharmacological effect of formononetin was studied extensively (Dutra et al., 2021).

However, the poor water solubility of formononetin strongly restricts its practical application. Glycosylation, one of the most popular modifications for natural products, was chosen to improve the water solubility of formononetin–ononin and its derivatives. It has been reported that the natural quercetin is biotransformed into isoquercitrin with a higher bioavailability and more chemoprotective effects (Rüfer et al., 2008; Chu et al., 2014). The water solubility and bioavailability of flavonoid aglycones were increased by glycosylation modification. The separation of ononin from plants is expensive and complicated, accompanied by a large number of toxic solvents (Feng et al., 2013). Generally, bacteria exhibited various biotransformation abilities, and succinylation is another important glycosylation modification (Zhang et al., 2011). *Bacillus* was first applied for triterpenoid glycoside succinylation by Chang et al. (2018). The formation of isoflavone in succinyl form can be achieved when β-glucoside is consumed by *Bacillus*, which is a great activity of glycosylation and succinylation (Chan et al., 2010). Biotransformation is an efficient route to produce important chemicals and can realize green production. However, many bacteria and their enzymes will be destroyed and inactivated in organic solvents, which limited the practical application of biotransformation in the chemical industry (Molina et al., 2010). A feasible solution is to use organic solvent-resistant bacteria as biocatalysts, which cannot only tolerate organic solvents but also has a high conversion rate (Wang et al., 2008). Our research group has developed the biosynthetic method of transforming formononetin into succinyl formononetin glycoside by organic solvent-resistant bacteria, which can improve the poor solubility and oral absorption (Zhang et al., 2016). *Bacillus amyloliquefaciens* FJ18 has been applied for efficiently catalyzing apigenin into succinyl-apigenin in non-aqueous phase. At present, the protective effect of formononetin against myocardial ischemia injury has been proven by some groups, and its mechanism has been explored (Wang et al., 2020), but the related mechanism of FMP has not been reported.

In this study, the method of bioconversion of formononetin into FMP by *Bacillus amyloliquefaciens* FJ18 was developed, which efficiently solved the limitation on water solubility and oral availability. Moreover, the bioactivity of formononetin was also investigated on myocardial protection *in vitro* (Gu et al., 2021; Machado Dutra et al., 2021). The protective effect of biosynthetic succinyl onion on acute myocardial ischemia induced by isoproterenol in mice was evaluated.

## Materials and Methods

### Materials

Formononetin was prepared in our laboratory. The solvents used in high-performance liquid chromatography (HPLC) analysis were of HPLC grade and obtained from Sigma. Other solvents and reagents were of analytical grade and purchased from commercial sources. The assay kits for lactate dehydrogenase (LDH), superoxide dismutase (SOD), and catalase (CAT) were purchased from Nanjing Jiancheng Institute of Biological Engineering. Isoproterenol was purchased from Sigma Company (propranolol; batch number: H2811226).

### Screening and Identification of Strains

One gram of soil sample was taken into a triangular flask containing a 20-ml screening medium, cultured in a water bath at 30°C and 180 r/min for 24 h, and then transferred three times with a 5% inoculation amount. After proper dilution, the culture broth was diluted and coated with LB plate culture medium, cultured at 30°C for 24 h, and the colony morphology was observed. Then a single colony was selected and separated on LB plate culture medium to obtain monoclonal, and then the screened strains were numbered and stored at ultra-low temperature. The preserved strain was inoculated into an LB plate culture medium from a freezing tube, cultured at 30°C for 24 h, then inoculated into screening culture medium (40-ml culture solution/250-ml triangular flask), cultured at 30°C for 24 h at 200 rpm. In this way, the fluorescence changes in the culture medium were observed, and then samples were taken for 0, 12, and 24 h, respectively, for HPLC (Zhang et al., 2016).

### Microbial Fermentation

The fermented screened strains was inoculated into seed culture medium: yeast extract 5.0 g/L, peptone 10.0 g/L, NaCl 10.0 g/L, pH 7.0, and cultured at 30°C and 200 rpm for 12 h. The expansion medium and fermentation medium are composed of sucrose 20 g/L, yeast powder 15 g/L, KH_2_PO_4_ 1.0 g/L, and CaCl_2_ 0.8 g/L. The pH was adjusted to 8.0 with NaOH 0.5% (v/v). Seed solution was inoculated into the expanded medium and fermentation medium, and cultured at 30°C and 200 rpm for 12 h. After centrifugation at 10,000 rpm for 15 min, the bacterial cells were collected and washed with normal saline one to two times to obtain resting cells of the fermentation strain (Zhang et al., 2016).

### Extraction of Formononetin and Ononin Derivatives

The product was separated by macroporous resin, and a proper amount of resin was taken and soaked in ethanol for 24 h to remove the resin fragments and impurities, packed in a wet column (Ø3 cm × 100 cm), washed with 1 L of ethanol, and then washed with distilled water until there is no smell of alcohol. Acid–base treatment was carried out in which 5% HCl solution by volume and 2% NaOH solution by mass, respectively, were passed through a resin column at a flow rate of 2 BV/h, made to stand still for 2–4 h, and then wash with distilled water until the pH is neutral. To prevent DMSO from dissolving the conversion product and reducing the adsorption rate, the conversion solution was diluted five times with deionized water (pH 4.0, adjusted by glacial acetic acid) until DMSO < 2%, and then the sample was added. Wet resin (20 mg/g) was added at an adding flow rate of 20 ml/min. The unreacted sugar-based donor (sucrose) was rinsed 10 times with deionized water (pH 4.0, adjusted by glacial acetic acid) until no sugar can be detected in the eluate with concentrated sulfuric acid at a flow rate of 20 ml/min. Methanol and deionized water were selected as mobile phases for elution, and the volume ratio of methanol and deionized water was adjusted. The ratio of methanol in the eluent was determined at a flow rate of 20 ml/min. After HPLC detection, the eluents were combined, and then concentrated under reduced pressure and vacuum with a rotary evaporator at a heating temperature of 40°C. Finally, the solid was put into a vacuum drying oven and dried at 40°C for 6 h. ^1^H NMR and ^13^C NMR spectra of the product were recorded in DMSO-d_6_ with a Bruker AV-400 spectrometer (Bremen, Germany) at 400 MH_Z_. HMBC experiments were also performed.

### Water Solubility of Formononetin and Ononin Derivatives

The calibration curve was established by HPLC, and the solubility of saturated aqueous solutions of formononetin, ononin, and FMP was determined at 37° (Guang-Jie et al., 2010).

### Cytotoxicity Test

Human normal myocardial cell H9C2 was selected as the research object for MTT assay, formononetin, ononin, and FMP were used, and each compound had three quantities to be measured (0.022, 0.066, and 0.200 μM). H9C2 cells in logarithmic growth phase were inoculated in a 96-well plate at a concentration of 5 × 10^4^ cells/ml and then cocultured with test compounds for 24 h. Then it was incubated for 4 h at 37°C with 20 μl of MTT solution. Cell viability was measured at 490 nm.

#### Animals

Seventy BALB/c mice (half were males and half were females), weighing 22–25 g, were provided by Shanghai Slack Experimental Animal Co., Ltd. All animal studies complied with the ARRIVE (Animal Research: Reporting of *In Vivo* Experiments) guidelines, and all the experimental protocols were approved by the Ethics Committee of Animal Experiments of Nanjing University of Chinese Medicine [SYXK(S)2018-0049, Nanjing, China]. H9C2 was purchased from Shanghai Cell Research Institute.

### Acute Ischemic Injury Model

A myocardial ischemia injury model with isoprenaline was established (Li et al., 2008). Seventy specific pathogen-free BALB/c mice (20–22 g) (half were males and half were females), were used for animal experiments. The mice were randomly divided into seven groups: blank control group, model group, positive drug group, formononetin (20 mg/kg) group, FMP high-, medium-, and low-dose groups (40, 20, and 10 mg/kg), with 10 mice in each group, fed adaptatively for 4 weeks. Mice were given intragastric administration for 10 days, while the blank group and model group were given normal saline and propranolol (20 mg/kg) 100 μl/10 g. Isoproterenol (10 mg/kg) was injected intraperitoneally on the 8th, 9th, and 10th day of administration. After the last administration for 2 h, orbital blood was collected, the hearts were taken out immediately for cryopreservation, and the heart tissue stained with HE was fixed and preserved with 4% paraformaldehyde.


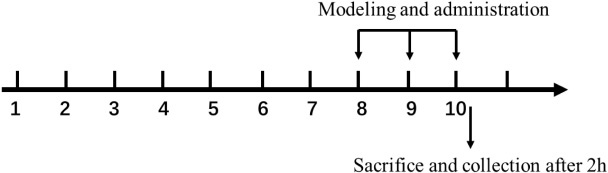


### Lactate Dehydrogenase, Superoxide Dismutase, and Catalase Test in Serum

After collecting blood from the mouse orbit, it was centrifuged at 4°C and 1,000 × *g* for 10 min. The serum was taken, and the related redox indexes were measured. The specific operation was carried out according to the kit instructions.

### Hematoxylin–Eosin Staining

Hematoxylin–eosin (HE) staining was performed on the heart to observe the changes in myocardial tissue after heart tissues were immersed with 4% paraformaldehyde for at least 24 h. Then the tissues were paraffin embedded, cut into 5-μm sections, and stained with H&E by standard histological techniques. Images were taken using a Leica DM 4000B photomicroscope. As previously reported, the severity of histological changes was determined using a five-point score system as follows (Table 1).

**TABLE 1:** The severity of histological changes.

Score	Severity of histological changes
0	Normal
1	Minimal
2	Mild
3	Moderate
4	Moderately severe
5	Severe

### Statistical Analysis

The data were calculated and expressed by SPSS22.0 for statistical analysis, and data are expressed as mean ± SD. Statistical analysis was performed with GraphPad Prism8.01 software according to Student’s *t*-test. The probability values of *p* < 0.05 were considered statistically significant.

## Results

### Selection of FJ18 for Biotransformation of Formononetin

*Bacillus amyloliquefaciens* FJ18 (Zhang et al., 2016), WJ02, ZSP01 (Zhang et al., 2015), and JQ06 were found to be organic solvent-resistant strains with the ability of formononetin biotransformation. FJ18 is capable of modifying formononetin by glycosylation and ononin by succinylation. However, the obtained succinyl ononin product is single. The transformation rate of formononetin by this strain reached 94.2% after optimization.

Formononetin and sucrose were chosen as raw materials; the 7-phenolic hydroxyl group of formononetin was glycosylated to form formononetin-7-*O*-glucoside after the catalysis of FJ18 in 10% DMSO. Formononetin-7-*O*-glucoside was succinylated at the 6-hydroxyl group of the glucose group to form FMP after the catalysis of FJ18. The reaction chemical formula is as follows:


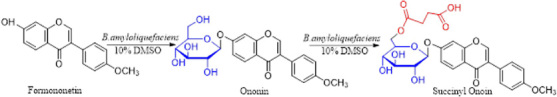


### Isolation and Characterization of Formononetin-7-*O*-β-(6″-*O*-Succinyl)-D-Glucoside

According to the NMR spectrum, the obtained structure is consistent with the expected product. The above results confirmed that glucosylated formononetin, namely, FMP, was produced in this reaction. The NMR spectrum data of formononetin-7-*O*-β-(6″-*O*-succinyl)-D-glucoside are shown in Table 2.

**TABLE 2:** ^1^H (300 MHz) and ^13^C (75 Hz) NMR data of formononetin-7-*O*-β-(6″-*O*-succinyl)-D-glucoside in DMSO-d6.

Position	δ^13^X	δ^1^H (J in Hz)
2	153.6	8.39 (1H, s)
3	124	
4	174.7	
5	127	8.07 (1H, d.8.9)
6	115.6	7.15 (1H, dd, 8.9, 2.4)
7	161.2	
8	103.5	7.24 (1H, d, 2.3)
9	157	
10	118.6	7.52 (2H, d.8.6)
1′	123.4	7.00 (2H, d, 8.6)
2′	130.1	
3′	113.7	7.00 (2H, d, 8.6)
4′	159.1	7.52 (2H, d.8.6)
5′	113.7	5.15 (1H, d, 7.4)
6′	130.1	
1″	99.8	5.15 (1H, d, 7.4)
2″	73.1	
3″	76.6	
4″	69.9	
5″	74	
6″	63.6	4.42 (1H, dd, 11, 2.4), 4.04
1″′	172	(1H, dd, 11, 7.2)
2″′	28.7	
3″′	28.7	2.47–2.57 (4H, m)
4″′	173.4	2.47–2.57 (4H, m)
OCH_3_	55.2	3.39 (3H, s)

### Water Solubility and Toxicity of Formononetin-7-*O*-β-(6″-*O*-Succinyl)-D-Glucoside

The solubility of formononetin, ononin, and FMP is 0.00208, 0.0125, and 0.221 g/L, respectively. Compared with formononetin and ononin, the solubility of FMP was increased over 106 times and 17 times, respectively.

**FIGURE 1:**
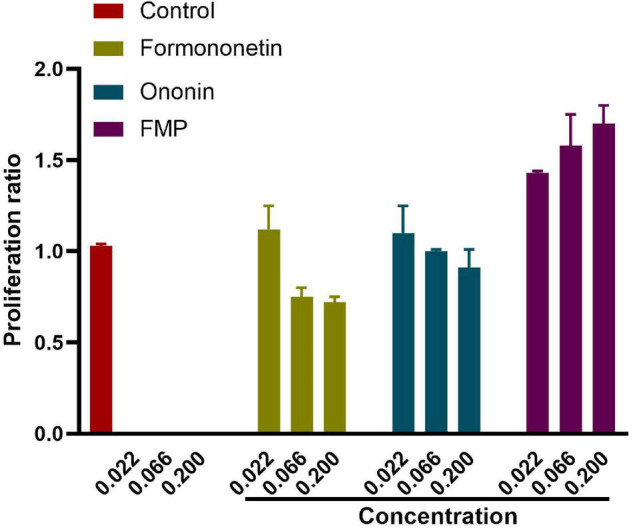
Effects of the different doses of formononetin, ononin, and formononetin-7-*O*-β-(6″-*O*-succinyl)-D-glucoside (FMP) on the growth of cardiac myocytes H9C2 (*n* = 10).

The results showed that formononetin and ononin inhibited H9C2 cells at medium and high doses (0.066, 0.2 μM). FMP showed a certain cytoprotective effect, but without cytotoxicity under the three test doses. Therefore, the toxicity of FMP to cardiac myocytes H9C2 is much lower than that of formononetin and ononin (Figure 1).

### Effects of Formononetin-7-*O*-β-(6″-*O*-Succinyl)-D-Glucoside on Lactate Dehydrogenase, Superoxide Dismutase, and Catalase Activity in Serum of Iso-Induced Myocardial Ischemia Mice

**FIGURE 2:**
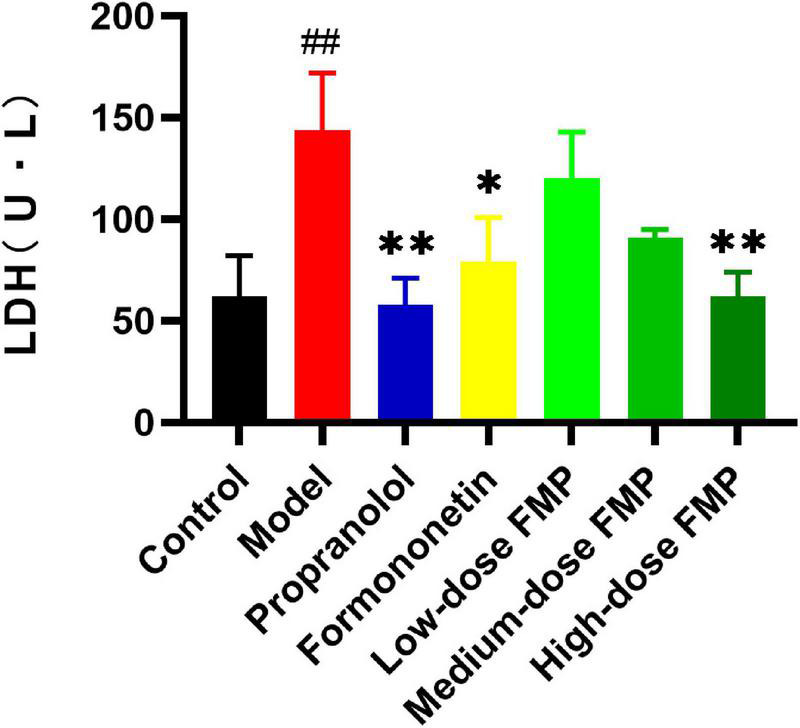
Effect of FMP on LDH activity in the serum of iso-induced myocardial ischemia mice (##*p* < 0.01 vs. control group; **p < 0.01; *p < 0.05 vs. model group).

Compared with the control group, the LDH activity in the serum of the model group was significantly increased (*p* < 0.01), which indicated that the model was successfully established. Compared with the model group, positive drugs and aglycones can significantly reduce LDH activity (*p* < 0.01, *p* < 0.05). FMP can also reduce LDH activity, but it has a significant difference only at a high dose (*p* < 0.01; Figure 2).

**FIGURE 3:**
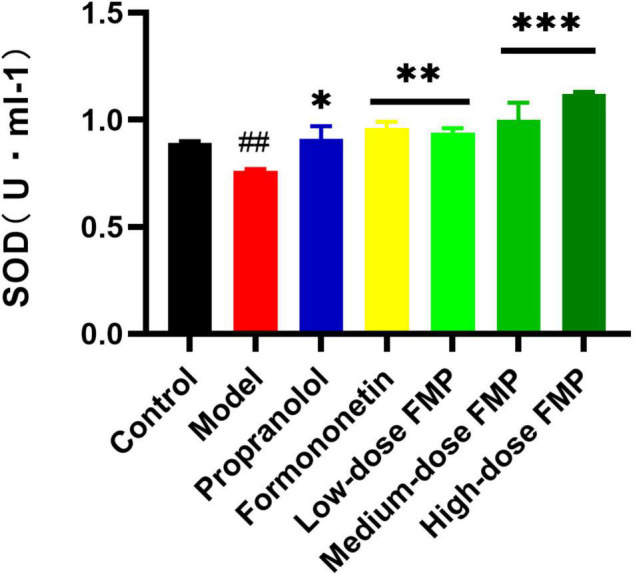
Effect of FMP on SOD activity in the serum of iso-induced myocardial ischemia mice (^##^*p* < 0.01 vs. control group; ***p < 0.001, **p < 0.01, *p < 0.05 vs. model group).

Compared with the control group, the activity of SOD in the serum of the model group decreased significantly (*p* < 0.01), while the SOD activity in the positive group and aglycones group increased significantly (*p* < 0.05, *p* < 0.01). As shown in Figure 3, the SOD activity in the serum of mice increased significantly (*p* < 0.01, *p* < 0.0001) after FMP administration.

**FIGURE 4:**
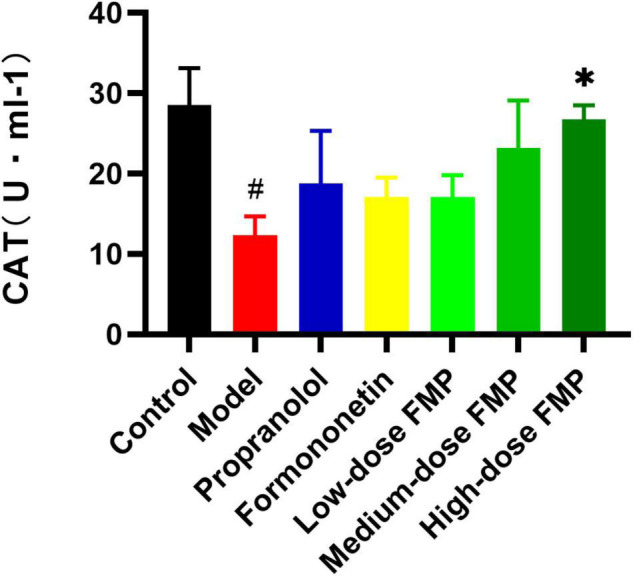
Effect of FMP on CAT activity in the serum of iso-induced myocardial ischemia mice (^#^*p* < 0.05 vs. control group; *p < 0.05 vs. model group).

Compared with the control group, the activity of CAT in the model group decreased significantly (*p* < 0.05). Compared with the model group, both the CAT activity of positive drugs and aglycones increased, but the difference was not statistically significant. After FMP administration, the activity of CAT increased, and FMP at a high dose could significantly increase CAT activity (*p* < 0.05; Figure 4). From Table 3, the LDH, SOD, and CAT activities showed that FMP is superior to formononetin, while formononetin is slightly superior to ononin.

**TABLE 3:** Effects of formononetin-7-*O*-β-(6″-*O*-succinyl)-D-glucoside (FMP) on SOD, LDH and CAT activities in serum of mice with myocardial ischemia ($\bar{\text{x}}\pm \text{s}$, *n* = 10).

Groups	Dose (mg⋅kg^–1^)	SOD (U ml^–1^)	CAT (U ml^–1^)	LDH (U L)
Blank control	–	0.89 ± 0.01	28.5 ± 4.6	62 ± 20
Model	–	0.76 ± 0.01^##^	12.3 ± 2.4^#^	144 ± 28^##^
Propranolol	20	0.91 ± 0.06*	18.8 ± 6.5	58 ± 13**
Formononetin	20	0.96 ± 0.03**	17.1 ± 2.4	79 ± 22*
Ononin	20	0.95 ± 0.02**	16.8 ± 2.1	75 ± 24
Low-dose FMP	10	0.94 ± 0.02**	17.1 ± 2.7	120 ± 23
Medium-dose FMP	20	1.00 ± 0.08***	23.2 ± 5.9	91 ± 4
High-dose FMP	40	1.12 ± 0.10***	26.7 ± 1.8*	62 ± 12**

^##^p < 0.01, ^#^p < 0.05 vs. control group; ***p < 0.001, **p < 0.01, *p < 0.05 vs. model group.

Formononetin showed higher bioavailability over ononin, which is an active metabolite of formononetin (Luo et al., 2018). The SOD activity of formononetin in PC12 cells is slightly superior to ononin (Yu et al., 2005). Our results showed that propranolol, formononetin, and FMP could improve myocardial tissue injury in mice with myocardial ischemia. Meanwhile, high-dose FMP significantly increased the activities of CAT and SOD and remarkably decreased the activity of LDH.

### Effect of Formononetin-7-*O*-β-(6″-*O*-Succinyl)-D-Glucoside on Pathomorphology of Myocardial Tissue in ISO-Induced Myocardial Ischemia

**FIGURE 5:**
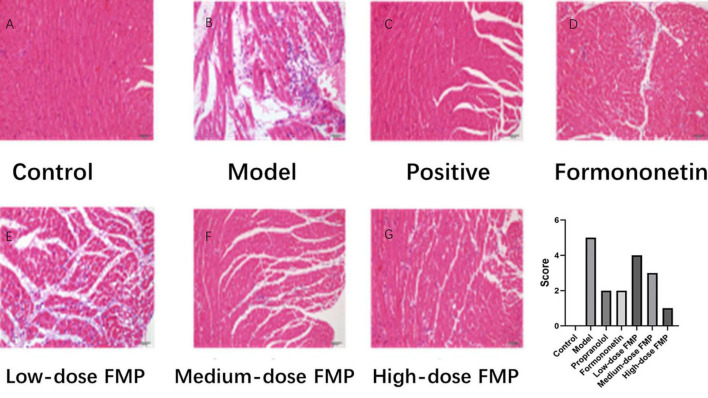
Effect of FMP on the pathomorphology of myocardial tissue. **(A)** Blank control group, **(B)** model group, **(C)** positive drug group, **(D)** glycogen group, **(E)** low-dose FMP, **(F)** medium-dose FMP, and **(G)** high-dose FMP.

Under a light microscope, the myocardial fibers in the blank control group were arranged neatly, with clear transverse stripes and obvious nucleus, without edema and inflammatory cell infiltration. In the model group, the myocardial tissue was obviously damaged, the myocardial cells were vacuolated, the striations disappeared, the nuclei moved inward, and sheet necrosis, severe inflammatory cell infiltration, and myocardial interstitial edema were discovered. Compared with the model group, FMP can obviously repair myocardial injury, observing the transverse stripes and significantly improving interstitial edema. Mild myocardial interstitial edema and a small amount of inflammatory cell infiltration can be observed in the myocardial tissue of mice. Especially, the improvement of myocardial ischemia injury is more noticeable in the high- and medium-dose groups. As shown in Figure 5, the effect of FMP on the pathomorphology of myocardial tissue in iso-induced myocardial ischemia mice (×200) was presented.

## Discussion

According to the previous laboratory study, FJ18, which was screened from an extreme environment, exhibited high transformation ability. The 7-phenolic hydroxyl group of formononetin is glycosylated to form formononetin-7-*O*-glucoside after the catalysis of FJ18, and then the hydroxyl group at the sixth position of glucosyl of formononetin-7-*O*-glucoside is succinylated to form FMP. Glycosidic reaction usually needs multistep operations, such as adding a protective group and deprotection. The generation of enantiomers of target products during reaction makes it difficult to separate and purify target products (Braemer et al., 1987). The biotransformation with FJ18 is simpler and more efficient than other transformation methods, which can be better applied to industrialization. Therefore, the high stabilities to organic solvents of the FJ18 strain are the basis of non-aqueous enzymatic biotransformation and the high efficiency of biocatalysis.

In addition, FMP has a significant effect on myocardial ischemia injury. ISO belongs to catecholamine substances observed from the pharmacological experiment. FMP can excite the heart, enhance myocardial contractility, and speed up heart rate, which makes myocardial oxygen consumption increase obviously, causing myocardial relative ischemia and hypoxia, and then inducing acute myocardial ischemia in mice (Kong and Huang, 2008; Li et al., 2008). With a decrease in blood flow and an increase in myocardial oxygen consumption, the myocardial tissue is in a state of ischemia and hypoxia, and a large number of oxygen-free radicals are produced at the same time, which leads to damage of the myocardial cell membrane, increase in permeability, and increase in LDH leaked to serum. At the same time, a large number of oxygen-free radicals can directly or indirectly cause lipid peroxidation, which leads to the destruction of the cell membrane structure or the functional barrier of the cell membrane, and consumes free radical scavenging enzymes such as SOD and CAT in cells (Zhu et al., 2014). Therefore, myocardial damage can be judged by measuring the activities of LDH, SOD, and CAT. In this study, FMP can improve the myocardial damage of mice with myocardial ischemia by scavenging free radicals in the serum to improve the activities of CAT and SOD and significantly reduce the activities of LDH, thus, reducing the damage of cell membrane and maintaining the integrity of cell membrane during myocardial ischemia. However, the optimal dose of FMP for myocardial ischemia injury needs further study.

Furthermore, the incidence of COVID-19 infection in patients with acute ischemic stroke was 46.3%, while that in controls was only 18.3%, which has shown that COVID-19 is an independent risk factor for acute ischemic stroke (Li, 2020). Thus, FMP is a promising candidate to treat myocardial ischemia injury caused by COVID-19.

The results of this study show that FMP has a protective effect on ISO-induced acute myocardial ischemia. However, the specific protective mechanism and the dosage of FMP need to be further studied. Therefore, our study provides a basis for the preparation of cardiovascular drugs by FMP. In the future, we aim to explore the therapeutic effect of the FMP on myocardial injury caused by COVID-19.

## Conclusion

With efficient succinylation of formononetin by *Bacillus amyloliquefaciens* FJ18, FMP was proven to be a new compound by ^1^HNMR, ^13^CNMR, and HMBC. This transformation significantly improved the water solubility of formononetin by 106 times and remarkably enhanced the bioavailability. Furthermore, this transformation avoided separating ononin and derivatives with high toxicity, and provided the possibility of applying it to clinical treatment. After the establishment of ISO-induced acute ischemic injury model, myocardial tissue injury was obvious, the activity of LDH in serum increased, and the activity of CAT and SOD decreased significantly. Compared with the model group, FMP can significantly improve myocardial injury, decrease LDH activity, and increase CAT and SOD activity. The pathomorphological study of myocardial ischemia shows that medium- and high-dose treatment of FMP can significantly repair myocardial injury, although the specific dose still needs to be explored.

## Data Availability Statement

The original contributions presented in the study are included in the article/Supplementary Material, further inquiries can be directed to the corresponding authors.

## Ethics Statement

The animal study was reviewed and approved by the animal committee of Nanjing University of Chinese Medicine.

## Author Contributions

LZ conceived of the study and wrote the manuscript. LZ, JL, KF, and JuH carried out the sample collection, data analysis, and the results validation. LC drafted the manuscript. SZ and TY provide the herbal extraction residues resources. JiH helped to revised the manuscript. All authors read and approved the final manuscript.

## Conflict of Interest

The authors declare that the research was conducted in the absence of any commercial or financial relationships that could be construed as a potential conflict of interest.

## Publisher’s Note

All claims expressed in this article are solely those of the authors and do not necessarily represent those of their affiliated organizations, or those of the publisher, the editors and the reviewers. Any product that may be evaluated in this article, or claim that may be made by its manufacturer, is not guaranteed or endorsed by the publisher.
