# Supplementary figures and images for: A Novel Formononetin Derivative Promotes Anti-ischemic Effects on Acute Ischemic Injury in Mice
Source: Front Microbiol. 2021 Dec 14;12:786464. doi: 10.3389/fmicb.2021.786464 (PMC8712702; doi:10.3389/fmicb.2021.786464)

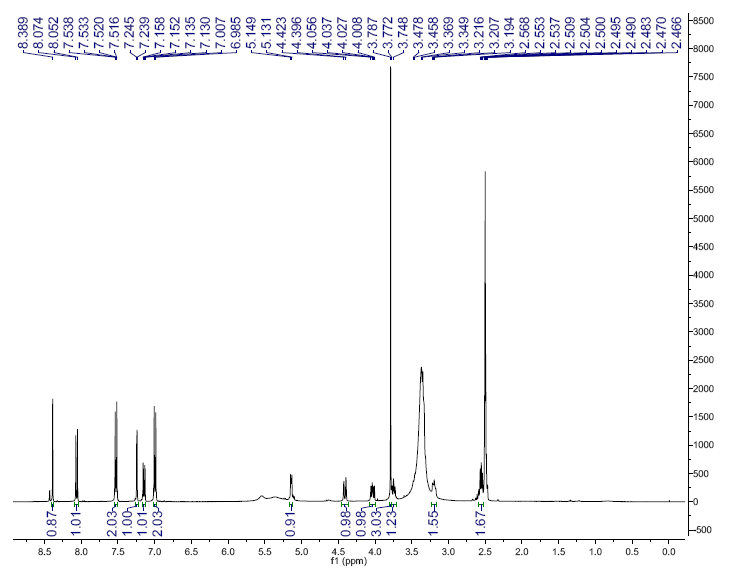


**Fig.S1**

**Fig.S2**

**Fig.S3**

Supplement: Supplementary file 1 [file Data_Sheet_1.docx]
